# A nested reciprocal experimental design to map the genetic architecture of transgenerational phenotypic plasticity

**DOI:** 10.1093/hr/uhae172

**Published:** 2024-06-25

**Authors:** Jincan Che, Yu Wang, Ang Dong, Yige Cao, Shuang Wu, Rongling Wu

**Affiliations:** Center for Computational Biology, College of Biological Sciences and Technology, Beijing Forestry University, Beijing 100083, China; Center for Computational Biology, College of Biological Sciences and Technology, Beijing Forestry University, Beijing 100083, China; Beijing Institute of Mathematical Sciences and Applications, Beijing 101408, China; Center for Computational Biology, College of Biological Sciences and Technology, Beijing Forestry University, Beijing 100083, China; Center for Computational Biology, College of Biological Sciences and Technology, Beijing Forestry University, Beijing 100083, China; Beijing Institute of Mathematical Sciences and Applications, Beijing 101408, China; Center for Computational Biology, College of Biological Sciences and Technology, Beijing Forestry University, Beijing 100083, China; Beijing Institute of Mathematical Sciences and Applications, Beijing 101408, China; Yau Mathematical Sciences Center, Tsinghua University, Beijing 100084, China

## Abstract

Extensive studies have revealed the ecological and evolutionary significance of phenotypic plasticity, but little is known about how it is inherited between generations and the genetic architecture of its transgenerational inheritance. To address these issues, we design a mapping study by growing *Arabidopsis thaliana* RILs in high- and low-light environments and further growing their offspring RILs from each maternal light environment in the same contrasting environments. This tree-like design of the controlled ecological experiment provides a framework for analysing the genetic regulation of phenotypic plasticity and its non-genetic inheritance. We implement the computational approach of functional mapping to identify specific QTLs for transgenerational phenotypic plasticity. By estimating and comparing the plastic response of leaf-number growth trajectories to light environment between generations, we find that the maternal environment affects phenotypic plasticity, whereas transgenerational plasticity is shaped by the offspring environment. The genetic architecture underlying the light-induced change of leaf number not only changes from parental to offspring generations, but also depends on the maternal environment the parental generation experienced and the offspring environment the offspring generation is experiencing. Most plasticity QTLs are annotated to the genomic regions of candidate genes for specific biological functions. Our computational-experimental design provides a unique insight into dissecting the non-genetic and genetic mechanisms of phenotypic plasticity shaping plant adaptation and evolution in various forms.

## Introduction

Phenotypic plasticity, referred to as the production of multiple phenotypes by the same genotype in different environments, is an ecological process. It is now widely regarded as one of the fundamental mechanisms by which the organism copes with environmental change to evolve [1–8]. Phenotypic plasticity is shaped by the dynamic interplay between environmental factors and genes. Environmental influences modulate phenotype expression through the regulation of the gene’s epigenetic modifications, such as histone modifications, RNA interference, and DNA methylation [9–11]. Studying the genetic basis of phenotypic plasticity has received growing interest in the past three decades, especially in recent years with the advent of high-throughput genomic techniques [7, 12–17].

An essential step to predict how phenotypic plasticity shapes evolution includes the characterization of its transgenerational inheritance through genetic and nongenetic alterations. Many studies have begun to investigate the adaptive significance of transgenerational plasticity in various environments [18–21]. However, it is still not known what the transgenerational pattern of inheritance of phenotypic plasticity is, or if and how this transgenerational inheritance is governed by specific genes. In the current literature, no experimental and computational strategies have been integrated to reveal these fundamental questions regarding the unified genetic and nongenetic (epigenetic) mechanisms of phenotypic plasticity, despite their significance for understanding the origin of evolutionary novelties.

**Figure 1:**
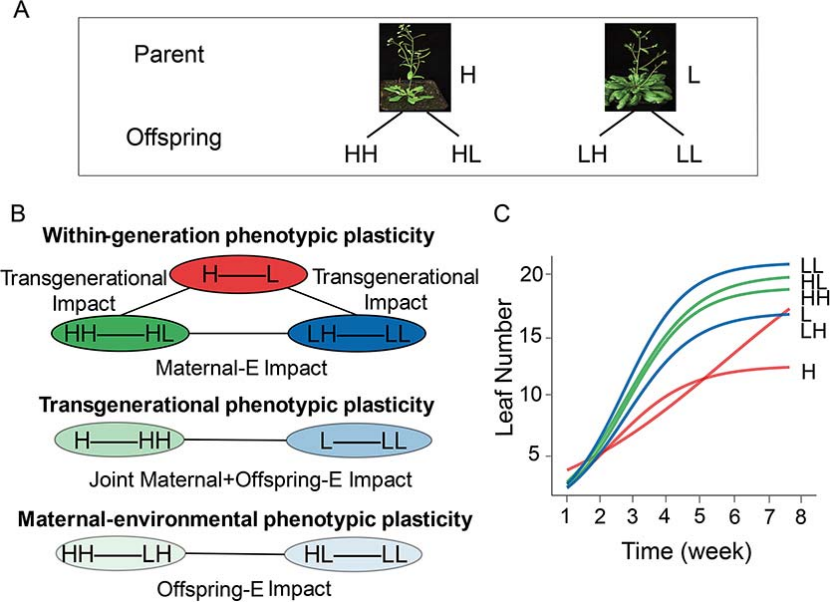
A reciprocal design of ecological experiment using *Arabidopsis*. **A** A panel of RILs derived from two grandparents are planted in a high-light (H) and low-light (L) treatment, respectively, and their offspring from the H maternal environment are planted in the H treatment (HH) and in the L treatment (HL) and from the L maternal environment planted in the H treatment (LH) and in the L treatment (LL). **B** Different forms of phenotypic plasticity, including within-generational, transgenerational, and maternal, each defined by the difference between a pair of relevant treatments. **C** The mean growth curves of leaf number over all RILs under six treatments, fitted by the logistic growth equation.

Here, we design an experiment in which a recombinant inbred line (RIL) panel of *Arabidopsis thaliana* offspring, derived from maternal high- and low-light environments, are respectively planted in the same high- and low-light conditions as their maternal environments (Fig. 1A). Here, we design an experiment in which a recombinant inbred line (RIL) panel of *A. thaliana* offspring derived from maternal high- and low-light environments are, respectively, planted in contrasting light conditions (Fig. 1A).

As a fuel for photosynthesis, light influences plant growth and development [22–24], widely used as an environmental factor to study plant phenotypic plasticity [25, 26]. We measure the grow trajectories of leaf number for each RIL from parental and offspring generations in high- and low-light conditions. This design allows us to investigate how the leaf phenotype of the same genotype varies between two environments within the same generation (within-generational phenotypic plasticity, WPP), between two generations under the same environment (transgenerational phenotypic plasticity, TPP), and between two maternally experiencing environments (maternal phenotypic plasticity, MPP) (Fig. 1B). By comparing the difference of WPP expressed in the offspring generation and its parental generation, we can test how phenotypic plasticity is inherited across generations. While the significance test of TPP informs us of how maternal environment influences offspring phenotypes, MPP is to describe the phenotypic plasticity of maternal environment. We performed statistical tests to determine the significance of differences in leaf number growth trajectories. These tests help us assess whether the observed differences are statistically significant, meaning they are unlikely to have occurred by chance.

A phenotypic trait can be better described by multiple values measured at different time points during plant development [27, 28]. The current suite of single-locus-based GWAS methods, while fundamental for exploring genome-wide associations between individual markers and phenotypes, may not be optimal for studying complex traits. Unlike static traits, developmental traits exhibit changes over time and in response to environmental factors. Hence, we can analyse their developmental process by constructing a functional map. This approach is based on mathematical models of organ development to map QTLs, thereby providing a better understanding of the spatiotemporal dynamics of individual traits [29–31]. Functional mapping has been leveraged to composite functional mapping (coFunMap) that can map a mathematical derivative of two or more traits [32]. Phenotypic plasticity is measured as the difference of trait values at a series of time points between two environments. As thus, we implement coFunMap to characterize the genetic architecture of developmental phenotypic plasticity. The resulting findings help us answer (i) whether maternal light environment influences offspring traits, (ii) whether there is genetic variation for maternal light effects, and (iii) whether this maternally induced genetic variation is different from that in intragenerational plasticity.

## Results

### Adaptive maternal environment

**Figure 2:**
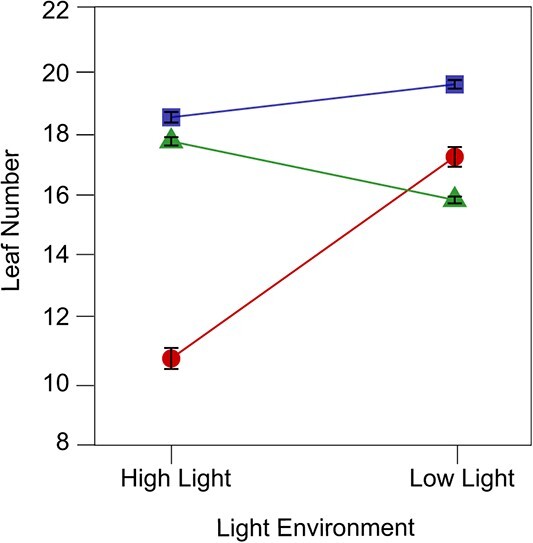
Asymptotic growth of leaf number (±SE) for RILs in six treatments. High Light and Low Light on the x axis denote the H and L maternal light environments, respectively. SE elucidates leaf number variation across RILs.

We use the logistic growth equation to fit the mean growth trajectories of leaf number for all RILs from each of six treatments, parental high light (H) vs. parental low light (L), offspring high light (HH) vs. offspring low light (HL) from parental H, and offspring high light (LH) vs. offspring low light (LL) from parental L (Fig. 1A). We find that there is pronounced variation in leaf number among RILs under each treatment (Fig. S1, see online supplementary material). Mean curves differ strikingly in growth rate and asymptotic leaf number among these treatments (Fig. 1C), including differences in growth trajectories between two light environments. We examine leaf numbers at the mature stage of plant development to investigate light-induced phenotypic plasticity (Fig. 2). As expected, plants develop more leaves in low light than in high light; for example, parental RILs grow seven more leaves in the low than high light (*P* < 0.05). We find that offspring RILs derived from a maternal low light develop four more leaves in the low light than in the high light (*P* < 0.01) and, also, offspring RILs from a maternal high light develop fewer leaves in the high than low light, despite being to a lesser significance level (*P* < 0.05). Taken together, the offspring tend to perform similarly to their parents in an environment that is the same as that which their parents have experienced.

### Maternal effects on phenotypic plasticity

**Figure 3:**
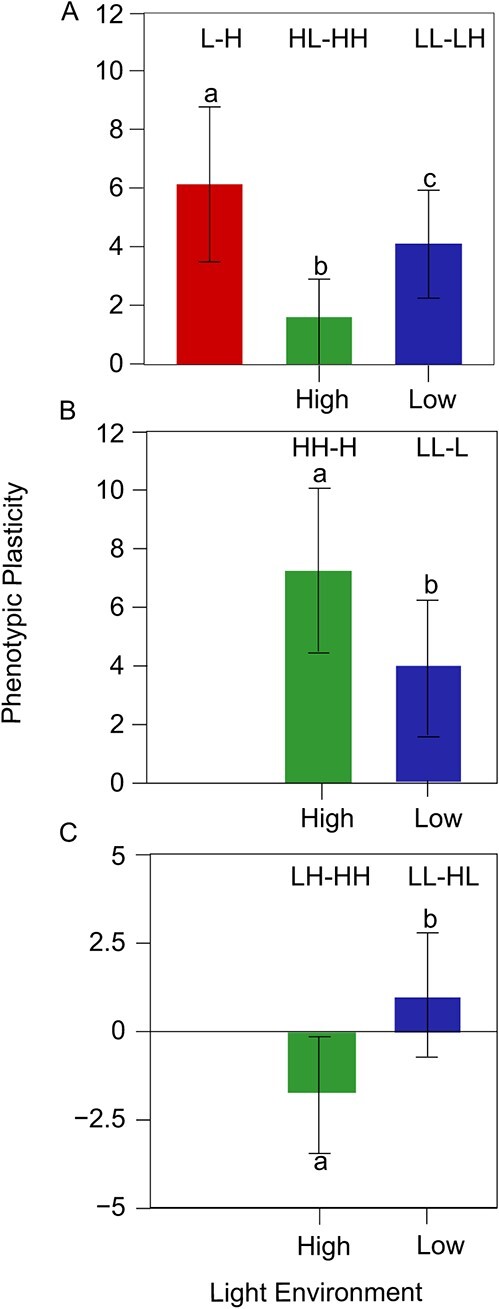
Three forms of phenotypic plasticity of asymptotic leaf number growth expressed as the difference between a pair of relevant treatments. **A** Mean within-generational phenotypic plasticity (±SE), displayed in the parental generation (left bar), the offspring generation from the H maternal environment (middle bar), and the offspring generation from the L maternal environment (right bar). **B** Mean transgenerational phenotypic plasticity (±SE), inherited from the maternal H environment (left bar) and the maternal L environment (right bar). **C** Mean maternal phenotypic plasticity formed in the H maternal environment (left bar) and in the L maternal environment (right bar), each displayed between the H and L offspring environments. Different letters on the bars indicate the significance of difference between the bars. SE elucidates leaf number variation across RILs.

The merit of our reciprocal design (Fig. 1A) lies in its capacity to disentangle different forms of phenotypic plasticity (Fig. 1B) and their respective contributions to plant adaptation and evolution. Depending on its origin, phenotypic plasticity can be classified into three forms. The first is WPP (within-generational phenotypic plasticity) that occurs within the same generation. There are two sub-forms of WPP, one occurring within the parental generation (pWPP = L – H) and the other within the offspring generation (oWPP involving oWPP_H = HL – HH and oWPP_L = LL – LH). If oWPP is different from pWPP, then this implies that maternal environment has an influence on phenotypic plasticity. As illustrated in Fig. 3A, we identify a pronounced influence of maternal light environment on the sensitivity of offspring’s leaf number to light environment. A reduced magnitude of oWPP_H and oWPP_L, compared to pWPP, suggests that phenotypic plasticity of leaf number is sensitive to the change of maternal light environment. It is also interesting to find that the magnitude of oWPP is dependent on maternal environment, with maternal low-light environment producing larger phenotypic plasticity than maternal high-light environment, i.e., oWPP_L > oWPP_H (*P* < 0.05) (Fig. 3A). This implies that offspring from a more favorable maternal environment tend to be more stable compared to those from a less favorable maternal environment.

The second form of phenotypic plasticity occurs between different generations, called TPP (transgenerational phenotypic plasticity) (involving TPP_H = HH – H and TPP_L = LL – L) (Fig. 3B). If TPP is significant, this means that maternal environment has an influence on offspring phenotypes. We find that TPP in leaf number is significant (*P* < 0.01) and positive in sign, suggesting that, by developing and transmitting a ‘memory’, maternal light environment promotes the leaf growth of offspring, expected to enhance their adaptability in the same environmental condition. The strength of this promotion depends on the type of maternal environment, with a larger strength by maternal high-light environment than by maternal low-light environment (*P* < 0.01). This suggests that a more favorable maternal environment can increase offspring leaf number (and, therefore, its correlated fitness [33]) to a larger extent than a less favorable maternal environment. The third form is expressed as MPP (maternal phenotypic plasticity) including MPP_H = HH – LH and MPP_L = LL – HL (Fig. 3C). The former two forms describe how and how much maternal environment affect offspring performance and phenotypic plasticity, whereas MPP informs the phenotypic plasticity of maternal environment. We find that MPP in leaf number is significant (*P* < 0.05), suggesting that maternal environment is a determinant of offspring leaf traits. In a more favorable high-light environment of the offspring, maternal environment is more plastic and, therefore, produces larger leaf number variation than in a less favorable low-light environment (*P* < 0.05).

### The genetic mechanisms of phenotypic plasticity

**Figure 4:**
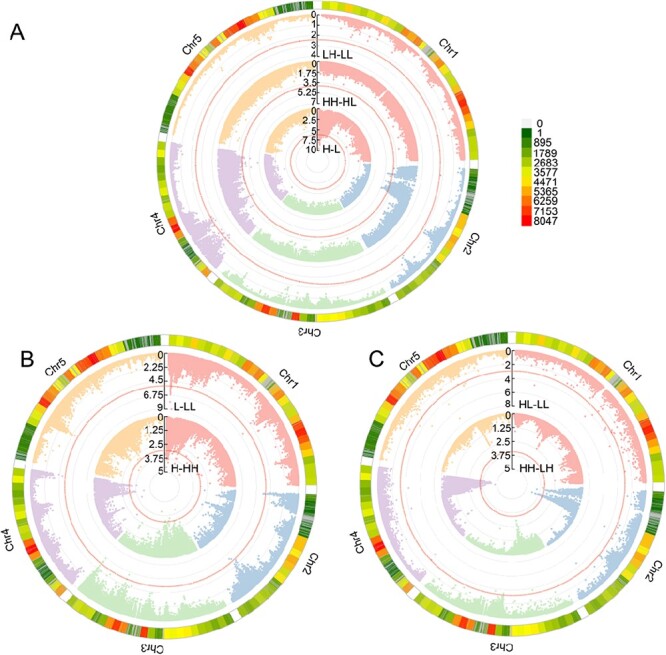
Cyclic Manhattan plots of P values for phenotypic plasticity through the whole Arabidopsis genome (composed of five chromosomes represented by distinct colors) calculated by coFunMap. **A** Within-generational phenotypic plasticity displayed in parental (inner cycle) and offspring generations (two outer cycles). **B** Transgenerational phenotypic plasticity in the H (inner cycle) and L maternal environment (outer cycle). **C** Maternal phenotypic plasticity expressed in the H offspring environment (inner cycle) and the L offspring environment (outer cycle). The chromosomal distribution of SNPs is denoted by a colored metric.

By viewing developmental phenotypic plasticity as a growth trait, we use coFunMap to map QTLs for WPP, TPP, and MPP in leaf number (Fig. 4). We identify 84 QTLs, all of which are located in chromosome 1, for WPP at the parental generation, 81 QTLs, located in chromosomes 2 and 4, for WPP_H at the offspring generation, and 24 QTLs, all located in chromosome 4, for WPP_L at the offspring generation (Fig. 4A). We find that WPP QTLs are different not only between different generations, but also depending on maternal light environment.

We identify 50 QTLs, distributed in chromosomes 1, 3, 4, and 5, for TPP_H, and 34 QTLs, distributed in chromosomes 1, 2, and 3, for TPP_L (Fig. 4B). It appears that TPP QTLs are distributed more sporadically in the chromosomes than WPP QTLs. The type of TPP QTLs depends on maternal environment. We find 40 QTLs, distributed in chromosomes 2 and 4, for MPP_H, and 28 QTLs, distributed in chromosomes 1 and 2, for MPP_L (Fig. 4C).

**Figure 5:**
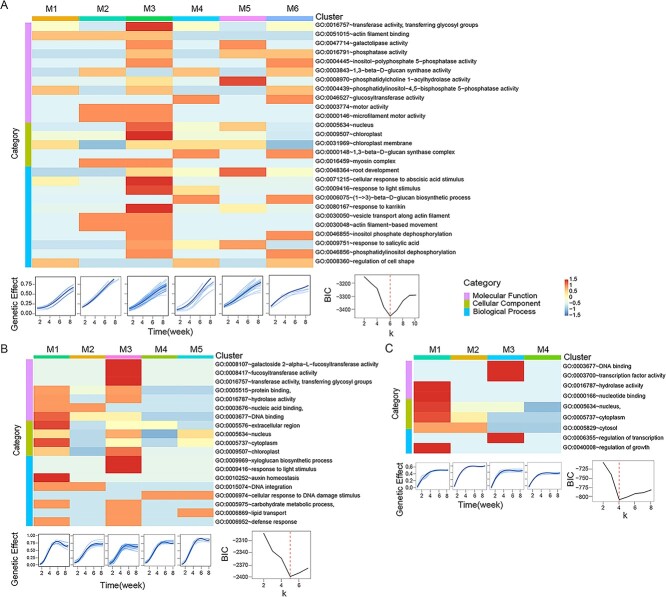
Modularity analysis of WPP QTLs. **A** Six QTL modules for pWPP including the heat map of gene functions, time-varying pattern of mean gene effects for each module, and the BIC plot finding an optimal number of QTL modules. **B** Five QTL modules for oWPP derived from the H maternal environment, including the heat map of gene functions, time-varying pattern of mean gene effects for each module, and the BIC plot finding an optimal number of QTL modules. **C** Four QTL modules for oWPP derived from the L maternal environment, including the heat map of gene functions, time-varying pattern of mean gene effects for each module, and the BIC plot finding an optimal number of QTL modules.

The overwhelming majority of QTLs do not overlap among these types of phenotypic plasticity (Fig. 5; Tables S1-S7, see online supplementary material), suggesting that plasticity QTLs are maternal environment-specific. We identify a number of QTLs in different genomic regions for phenotypic plasticity, which may function differently among modules. We implement functional clustering to sort all QTLs for each type of phenotypic plasticity into distinct modules based on their similarity of temporal genetic effects. We classify pWPP QTLs into six modules (Fig. 5A), oWPP_H QTLs into five modules (Fig. 5B), and oWPP_L QTLs into four modules. Curves of modules from pWPP QTLs and oWPP QTLs differ from one another, showing the impact of gene × maternal-environment interaction on phenotypic plasticity. Also, for the offspring generation, module curves of oWPP_H QTLs differ from those of oWPP_L QTLs, indicating the contribution of gene × maternal-environment × offspring-environment interaction to phenotypic plasticity. For the same sub-form of WPP, different modules exhibit distinct temporal patterns of genetic effects on leaf number, implying that genes from different modules perform different functions. We perform GO analysis of genes within the module and find that modules vary dramatically in terms of molecular functions, cellular components, and biological processes. Modules M1 and M3 are rich in genes encoding hydrolase activity and transferase activity important for plant growth, development and fruit ripening [64]. M1 and M3 also contain genes involved in photosynthesis and light stimulus response, typically located in chloroplasts. In general, pWPP contains different types of genes and functions from oWPP, and the same is true for oWPP_H vs. oWPP_L (Fig. 5).

**Figure S1:** 

**Figure S2:** 

**Figure S3:** 

**Figure S4:** 

We classify TPP_H QTLs into five modules (Fig. S3A, see online supplementary material) and TPP_L QTLs into five modules (Fig. S3B, see online supplementary material). We find that modules of TPP_H QTLs are very different from those of TPP_L QTLs. Curves of TPP_H modules are S-shaped, whereas curves of TPP_L modules display somewhat periodic shapes with time. Genes in a different module have specific functions. For example, Module 1 of TPP_L QTLs is rich in genes for glutathione binding and glutathione transferase activity, both of which are involved in the response mechanisms and phytoremediation of plants under various stresses [65, 66]. M1 also contains genes for glutathione binding and glutathione transferase activity, which may exert repairing and detoxifying functions in *Arabidopsis* leaves under low light stress, allowing *Arabidopsis* to better adapt to low light environment by increasing leaves number. We classify MPP_H QTLs into four modules (Fig. S4A, see online supplementary material) and MPP_L QTLs into four modules (Fig. S4B, see online supplementary material). Genetic effect curves of MPP QTLs differ strikingly from those of WPP QTLs and TPP QTLs. Also, the effect curves are different between MPP_H QTLs and MPP_L QTLs. It appears that inter-module differences in effect curve shape are more considerable for MPP_L QTLs than for MPP_H QTLs. GO analysis shows that each module from MPP_H QTLs or MPP_L QTLs contains a different set of genes that function differently in terms of molecular function, cellular component, and biological processes.

## Discussion

Phenotypic plasticity is influenced by a combination of factors including environmental conditions and regulation of gene expression (e.g., epigenetic regulation), which combine to determine an individual’s ability to adapt to environmental changes [9–11]. Growing evidence suggests that specific epigenetic modifications exist to serve as a mechanism driving the organism’s offspring to develop a ‘memory’ of their maternal environment in a quest to better adapt to the same environmental condition [11, 34–42]. The possibility of the existence of such maternally driven epigenetic marks leads us to characterize their underlying genetic basis as an essential step to improve our understanding of evolution. By specifying the processes of (i) how phenotypic plasticity is carried over from parental to offspring generations, (ii) at which level transgenerational plasticity is expressed, and (iii) whether the maternal ‘memory’ is environment-dependent, we want to map specific genes underlying each of them. To our best knowledge, our study here presents one of the most systematical characterization of genetic control over the transgenerational inheritance of phenotypic plasticity.

Our study was based on an ecological experiment by growing an RIL panel of *Arabidopsis Thaliana* in two contrasting environments and reciprocally growing the offspring from each maternal environment in the same set of two contrasting environments. The choice of *Arabidopsis Thaliana* as a research material stems from its unique advantages. Its RILs maintain genotype stability, making it one of the classic models for studying phenotypic plasticity. Additionally, its characteristics as a small model plant, rich genomic information, and diverse genetic resources provide abundant options for research. Thus, we can precisely quantify the difference of the same genotype between its parental and offspring generations, i.e., transgenerational plasticity. In summary, this design allows phenotypic plasticity to be defined across both time and space. On space, we define WPP at either parental or offspring generation, whereas across time, TPP is defined under each maternal environment. While TPP describes how maternal environment influences offspring traits and WPP describes how maternal environment influences the phenotypic plasticity of offspring traits, the third form of phenotypic plasticity, MPP, can characterize the phenotypic plasticity of maternal environment.

We find that the number of leaves follows different growth trajectories in low- and high-light conditions, with values being always higher in the former than in the latter. Under low-light conditions, *A. thaliana* may produce more leaves, possibly by upregulating light signaling factors such as the HY5 gene, to increase leaf numbers and enlarge leaf surface area for enhanced light capture, thereby compensating for reduced light intensity. Such a physiological response of plants to light factors is subject to maternal reprogramming. Offspring derived from the maternal light environment display a reduced magnitude of phenotypic plasticity (Fig. 2). However, light-induce response is much stronger for offspring derived from a low-light maternal environment than those from a high-light maternal environment. This difference is due to the fact that offspring derived from a low-light maternal environment grow much more leaves in a low light than in a high light, whereas offspring derived from a high-light maternal environment only grow slightly more leaves in a low light than in a high light. This finding suggests that offspring tend to produce a phenotype similar to their parents in the same environment experienced by the parents. In a previous study, Galloway [37] found that American tumbleweed grown in a maternal light environment had significantly higher seed sterilization rates. Progeny of plantain treated with parent nutrients accumulated more biomass and greater storage of root carbohydrates [43]. Our finding about leaf number plasticity in *Arabidopsis* gains additional insight into the function of maternally induced environment as an adaptive trait [38].

Currently, there are several methods for genome-wide association analysis, such as single-locus (sGWAS), metabolomics (mGWAS), gene expression (eGWAS), and haplotype (hGWAS) methods [44–46]. However, these approaches have limitations in deciphering the complete genetic mechanisms underlying complex traits. For traits exhibiting dynamic changes over time, such as biomass, root development, and leaf number, employing functional mapping methods incorporating time series functions may be more effective. Compared to previous genetic mapping of phenotypic plasticity, our study based on a reciprocal ecological design of a mapping population is more powerful in several aspects. First, our phenotypic plasticity is defined as being dynamic. Any biological trait can be better described by a developmental process. Functional mapping of developmental phenotypic plasticity has proven to be more powerful than static mapping approaches [32].

Second, our study allows the genetic architecture of WPP, TPP, and MPP, which can capture multifaceted features of phenotypic plasticity, to be disentangled under a unified framework. WPP describes how the organism responds to extrinsic environmental factors, such as light, temperature, or density. WPP differs not only between different generations (affected by the maternal environment), but also between the offspring environments. We found that there is little overlap in genetic control among different types of WPP (Fig. 4; Fig.S2, see online supplementary material). TPP specifies how the organism changes its phenotype in response to intrinsic generational environment. Many previous studies show that TPP is adaptive [18–21], and our study adds more knowledge, showing that the strength of TPP is environment-dependent. There is also little commonality for the QTLs for high light-exposed TPP and low light-exposed TPP (Fig. 4; Fig.S2, see online supplementary material). MPP is understood as the phenotypic plasticity of maternal environment and can also be understood as how maternal environment affects offspring performance. The strength of MPP is dependent on the environment the offspring is experiencing, which is controlled by a unique set of QTLs (Fig. 4; Fig.S2, see online supplementary material). Little overlap was detected for the QTLs affecting different forms of phenotypic plasticity, implying that various adaptive responses of the organism, subject to natural selection through divergent genetic and epigenetic systems, facilitate multiple avenues for producing diverse evolutionary novelties.

Third, we implement an advanced statistical model [47] to classify all plasticity QTLs into distinct modules, each with a different temporal pattern of genetic effects (Fig. 5; Figs. S3-S4, see online supplementary material). GO analysis helps us identify specific biological functions for each module; for example, some modules mediate chloroplasts and their photosynthesis, some mediate physiological responses to light and toxic substance, and some mediate phytoremediation. Taken together, tremendous genetic divergence among different forms of phenotypic plasticity and among different QTL modules may help the organism maintain its robustness to various environmental perturbations.

Perhaps the most significant merit of this study is to systematically characterize the impact of maternal environment on offspring traits and their phenotypic plasticity. Because plants lack a capacity to escape the change of their environment, they may enable maternal effects as a capacity to sense and perceive environmental cuing between generations to enhance offspring fitness [38]. Our detailed characterization of the genetic architecture underlying maternal environment provides a different dimension of understanding the evolutionary mechanisms by which sedentary organisms cope with heterogeneous environments. We find different genetic systems that mediate the phenotypic plasticity of offspring traits derived from high-light maternal and low-light maternal environments. Different genetic systems are also detected for transgenerational plasticity derived from these two maternal environments. We further find that the genetic architecture of maternal-environmental plasticity varies, depending on the offspring environment. Taken together, we identify high complexities of genetic control over maternal environment, which may explain why sedentary plants can adapt to a wide range of environmental fluctuations in the wild. A follow-up study by sequencing epigenetic marks can strengthen and validate our findings and interpretation about maternally induced phenotypic plasticity.

## Conclusion

As a capacity for plants to adapt to changing environment [1], phenotypic plasticity of ecological and evolutionary significance [4, 5, 48] has long been an important subject of plant biological research [2–5, 17, 25, 49–51]. More recently, the study of phenotypic plasticity has been expanded to biomedical fields where the change of health state can be explained through the underlying theory of phenotypic plasticity [52]. The genetic mapping of different forms of phenotypic plasticity by our design may not only inform plant researchers of how it is evolving, but also provide unique insight into studying the genetic basis of health-related plasticity.

## Materials and methods

### Reciprocal design of an ecological experiment

Plant materials used in this study are a RIL mapping population derived from 10 generations (F_10_) of self-fertilization between two *A. thaliana* ecotypes, *Landsberg erecta* (*Ler*) and *Shahdara* (*Sha*) [53]. This population contains 100 RILs, each of which was planted with 20 replicates under high- and low-light conditions laid out in an artificial climate chamber at Beijing Forestry University Greenhouse. We used standard containers and methods commonly employed for *A. thaliana* cultivation. Two chambers each with a different level of light were maintained at a constant temperature of 22°C and with adequate water and fertilization. The light conditions included a high light intensity of 141.2 μmol/(sm^2^) and a low light intensity of 89.8 μmol/(sm^2^), with the light duration set to 16 h/d. The number of leaves of each plant was measured from one week after planting for 8 consecutive weeks. These data collected were from RILs at the parental generation.

After *Arabidopsis* fruit pods were matured, we collected and mixed seeds of all individual plants from each RIL under each light condition. We transplanted seeds from each RIL, originating from distinct maternal light environments, into seedlings with 20 replicates per RIL under two light conditions, mirroring those used for the parental RILs (Fig. 1A). The same measure schedule of leaf number was taken to produce the data for RILs at the offspring generation. We averaged leaf numbers of 20 replicates per RIL as the trait value of this line per light treatment. Developmental phenotypic plasticity is quantified by the time-varying differences of traits value between different light environments, between different generations, and between different maternal environments.

### Genotyping

To genotype the panel of RILs, genomic DNA was extracted from leaves of each line using the TIANGEN DP305 kit. Sequencing was performed using Illumina sequencing technology. Raw sequence data quality was assessed using FastQC software, and reads were further processed and filtered for quality using fastp software to remove low-quality reads [54]. The *Arabidopsis* reference genome and annotation information were downloaded from the EnsemblPlants database. Using the available high-quality sample sequences and reference information from the TAIR website (https://www.arabidopsis.org/download/index-auto.jsp?dir=%2Fdownload_files%2FSequences%2FAssemblies, accessed on 1 October 2022), the reads of each sample were compared to the *Arabidopsis* reference genome with the bwa software to obtain the genome sequence of each lineage sample [55].

Variant calling was performed using SAMTOOLS software to detect single nucleotide polymorphisms (SNPs) at the population level. Subsequent SNP filtering was conducted using VCFtools based on predefined criteria. After processing, a total of 107 samples with 1 023 325 genome-wide SNP markers were obtained. Following exclusion of certain phenotypic and genotypic deletion lines, as well as removal of duplicate markers, 417 495 high-quality SNPs remained for further analysis. Detailed annotation of SNP loci, including chromosome, start position, genotype, locus function, and gene labeling information, was conducted using ANNOVAR software. Data analysis was then performed using both phenotypic and genotypic data collected during the experiment.

### Statistical and bioinformatics analysis

Phenotypic plasticity is measured as the phenotypic difference of a trait expressed in two distinct environments. Thus, mapping phenotypic plasticity is equivalent to mapping environment-induced trait differences. Unlike traditional mapping studies, our mapping model is based on time trajectories of phenotypic traits. Functional mapping (FunMap) combines the mathematical aspects of trait development into a mapping framework, allowing the QTLs for growth curves to be characterized [29, 31]. Sang *et al.* [32] extended FunMap to the case of a composite trait that is expressed as a mathematical function of two or more traits. This extended model, called coFunMap, was used to map all different forms of phenotypic plasticity of leaf number trajectories and find significant plasticity QTLs from a panel of genome-wide SNPs.

Let ***x****_i_* = (x_i_(t_1_), . . ., x_i_(t_T_)) and **y**_i_ = (y_i_(t_1_), . . ., y_i_(t_T_)) denote leaf numbers measured at a series of time points (*t_1_*, . . ., *t_T_*) for the *i*th RIL in two light environments X and Y, respectively. Developmental phenotypic plasticity is defined as the environment-dependent difference in the leaf number of the *i*th RIL over time, which is calculated as


(1) \begin{equation*} {\mathbf{z}}_{\mathrm{i}}=\left({\mathrm{z}}_{\mathrm{i}}\left({\mathrm{t}}_1\right),\dots, {\mathrm{z}}_{\mathrm{i}}\left({\mathrm{t}}_{\mathrm{T}}\right)\right)\equiv \left(\left({\mathrm{x}}_{\mathrm{i}}\left({\mathrm{t}}_1\right)-{\mathrm{y}}_{\mathrm{i}}\left({\mathrm{t}}_1\right)\right),\dots, \left({\mathrm{x}}_{\mathrm{i}}\left({\mathrm{t}}_{\mathrm{T}}\right)-{\mathrm{y}}_{\mathrm{i}}\left({\mathrm{t}}_{\mathrm{T}}\right)\right)\right) \end{equation*}

where the size and sign of each element at a specific time point reflect the degree and pattern of how the *i*th RIL responds to environmental change from X to Y over the time course. Consider a SNP *s* with *J_s_* genotypes. Let *n_js_* denote the observation of genotype *j_s_* (*j_s_ = 1, . . ., J_s_*). According to Sang *et al.* [32], we formulate a likelihood of coFunMap with developmental phenotypic plasticity at SNP s, expressed as


(2) \begin{equation*} {\mathrm{L}}_{\mathrm{s}}\left(\mathbf{z}\right)=\prod_{{\mathrm{j}}_{\mathrm{s}}=1}^{{\mathrm{J}}_{\mathrm{s}}}\prod_{\mathrm{i}=1}^{{\mathrm{n}}_{{\mathrm{j}}_{\mathrm{s}}}}{\mathrm{f}}_{\mathrm{s}}\left({\mathbf{z}}_{\mathrm{i}};{\boldsymbol{\mathrm{\mu}}}_{{\mathrm{j}}_{\mathrm{s}}},{\Sigma}_{\mathrm{s}}\right) \end{equation*}

where *f_s_*(·) is a *T*-dimensional normal distribution with mean vector *μ_js_* for genotype *j_s_* and residual covariance matrix Σ at SNPs. Based on the definition, the mean vector can be expressed as


(3) \begin{align*} \notag{\boldsymbol{\mathrm{\mu}}}_{{\mathrm{j}}_{\mathrm{S}}}& =\left({\mathrm{\mu}}_{{\mathrm{j}}_{\mathrm{s}}}\left({\mathrm{t}}_1\right),\dots, {\mathrm{\mu}}_{{\mathrm{j}}_{\mathrm{s}}}\left({\mathrm{t}}_{\mathrm{T}}\right)\right) \\{}& =\big({\mathrm{\mu}}_{{\mathrm{j}}_{\mathrm{s}}}^{\mathrm{x}}\left({\mathrm{t}}_1\right)-{\mathrm{\mu}}_{{\mathrm{j}}_{\mathrm{s}}}^{\mathrm{y}}\left({\mathrm{t}}_1\right),\dots, {\mathrm{\mu}}_{{\mathrm{j}}_{\mathrm{s}}}^{\mathrm{x}}\left({\mathrm{t}}_{\mathrm{T}}\right)-{\mathrm{\mu}}_{{\mathrm{j}}_{\mathrm{s}}}^{\mathrm{y}}\left({\mathrm{t}}_{\mathrm{T}}\right)\big)\notag\\{}& =\big({\mathrm{\mu}}_{{\mathrm{j}}_{\mathrm{s}}}^{\mathrm{x}}\left({\mathrm{t}}_1\right),\dots, {\mathrm{\mu}}_{{\mathrm{j}}_{\mathrm{s}}}^{\mathrm{x}}\left({\mathrm{t}}_{\mathrm{T}}\right)\big)-\big({\mathrm{\mu}}_{{\mathrm{j}}_{\mathrm{s}}}^{\mathrm{y}}\left({\mathrm{t}}_1\right),\dots, {\mathrm{\mu}}_{{\mathrm{j}}_{\mathrm{s}}}^{\mathrm{y}}\left({\mathrm{t}}_{\mathrm{T}}\right)\big) \end{align*}

where ${\mu}_{j_s}(t)$ is the mean phenotypic plasticity of SNP s genotype *j_s_* at time point *t* (*t* = 1, . . .., *T*), ${\mu}_{j_s}^x$and ${\mu}_{j_s}^y$ are the mean values of the leaves number of SNP s genotype *j_s_* at time point *T*, expressed in the X and Y environments, respectively. We consider that the time-dependent variation of leaf number in *Arabidopsis* follows an S-shaped curve described by a logistic equation [56–61]. Therefore, we modeled the genotypic mean of leaves number in each environment as


(4A) \begin{equation*} {\mathrm{\mu}}_{{\mathrm{j}}_{\mathrm{s}}}^{\mathrm{x}}\left(\mathrm{t}\right)={\mathrm{a}}_{{\mathrm{j}}_{\mathrm{s}}}^{\mathrm{x}}\Big[\big(1+{\mathrm{b}}_{{\mathrm{j}}_{\mathrm{s}}}^{\mathrm{x}}\cdotp \exp \big(-{\mathrm{r}}_{{\mathrm{j}}_{\mathrm{s}}}^{\mathrm{x}}\mathrm{t}\big)\Big]{}^{-1} \end{equation*}

(4B) \begin{align*} {\mathrm{\mu}}_{{\mathrm{j}}_{\mathrm{s}}}^{\mathrm{y}}\left(\mathrm{t}\right)={\mathrm{a}}_{{\mathrm{j}}_{\mathrm{s}}}^{\mathrm{y}}\Big[\big(1+{\mathrm{b}}_{{\mathrm{j}}_{\mathrm{s}}}^{\mathrm{y}}\cdotp \exp \big(-{\mathrm{r}}_{{\mathrm{j}}_{\mathrm{s}}}^{\mathrm{y}}\mathrm{t}\big)\Big]{}^{-1} \end{align*}

where growth parameters (*a, b, r*) are the asymptotic growth, the parameter that describes the initial growth, and the average specific growth rate of the curve, respectively, which are genotype- and environment-specific; i.e., two sets of growth parameters for each genotype are used for curve fitting for two different environments.

The statistical power of coFunMap also could be attributed to covariance modeling [31, 32, 62, 63]. The structure of the residual covariance matrix of developmental phenotypic plasticity is described below


(5) \begin{align*} &\notag{\boldsymbol{\Sigma}}_s\!=\!\left(\begin{array}{@{}ccc@{}}{\sigma}_s^2\left({t}_1\right)& \cdots & {\sigma}_s\left({t}_1,{t}_T\right)\\{}\vdots & \ddots & \vdots \\{}{\sigma}_s\left({t}_T,{t}_1\right)& \cdots & {\sigma}_s^2\left({t}_T\right)\end{array}\right)=\left(\begin{array}{@{}ccc@{}}{\sigma}_{sx}^2\left({t}_1\right)& \cdots & {\sigma}_{sx}\left({t}_1,{t}_T\right)\\{}\vdots & \ddots & \vdots \\{}{\sigma}_{sx}\left({t}_T,{t}_1\right)& \cdots & {\sigma}_{sx}^2\left({t}_T\right)\end{array}\right)\!+\\&\qquad\qquad\qquad\qquad \left(\begin{array}{@{}ccc@{}}{\sigma}_{sy}^2\left({t}_1\right)& \cdots & {\sigma}_{sy}\left({t}_1,{t}_T\right)\\{}\vdots & \ddots & \vdots \\{}{\sigma}_{sy}\left({t}_T,{t}_1\right)& \cdots & {\sigma}_{sy}^2\left({t}_T\right)\end{array}\right) \end{align*}

where the time-dependent variance ${\sigma}_s^2\left({t}_1\right)$ (and covariance ${\sigma}_s^2\left(t,{t}^{\prime}\right)$, t,t’ = 1, . . ., T) of phenotypic plasticity in the two settings is the sum of the temporal variances of ${\sigma}_{sx}^2(t)$ and ${\sigma}_{sy}^2(t)$ (and covariances ${\sigma}_{sx}\left(t,{t}^{\prime}\right)$ and ${\sigma}_{sy}\left(t,{t}^{\prime}\right)$), assuming that the two environments are independent of each other. The two environment-related covariance matrices in equation (5) contain longitudinal information; therefore, using an autoregressive model, such as the first-order structured antedependence SAD(1) model [32], to fit the structure of the covariance can increase the parsimony of the model. SAD(1) has the advantages of allowing the variance and covariance to vary over time without additional parameters and the existence of closed forms of matrix inverse and determinant that facilitate efficient computation.

To test whether a SNP is a significant QTL for developmental phenotypic plasticity, we tested two hypotheses [29, 32]. In the null hypothesis that there is no QTL, we estimate the likelihood value of the data assuming the existence of only a single mean curve. In the alternative hypothesis that there is a QTL, we estimate the likelihood value of the data under equation (2). The log-likelihood ratio (LR) under these two hypotheses is calculated as a test statistic, which is compared with the genome-wide critical threshold at 5% significance level determined from 100 permutation tests. The threshold is the 0.95 quantile of the LR values calculated from reshuffling data.

### Functional clustering

CoFunMap can estimate genotypic curves of leaf number at each plasticity QTL and further calculate and draw its genetic effect curves. We implemented functional clustering [47] to classify all plasticity QTLs into distinct modules in which QTLs from the same module are more similar in their temporal pattern to each other than to those from different modules. An optimal number of modules among all detected QTLs was determined according to BIC (Tables S8-S14).

If numerous QTLs are detected for phenotypic plasticity, we implement functional clustering to classify these QTLs into different modules in each of which QTLs follow a more similar temporal pattern of genetic effects than those from other modules. To do so, we first estimate the genetic standard deviations (GSD) of phenotypic plasticity of SNP *s* (*s* = 1, . . ., *S*) at any time point *t* using the maximum likelihood estimates (MLEs) of the growth parameters modeled for the mean vectors (4A) and (4B) calculated as


(6) \begin{equation*} {\mathrm{g}}_{\mathrm{s}}\left(\mathrm{t}\right)=\sqrt{\frac{1}{\mathrm{n}}\sum_{{\mathrm{j}}_{\mathrm{s}}=1}^{{\mathrm{J}}_{\mathrm{s}}}{\mathrm{n}}_{{\mathrm{j}}_{\mathrm{s}}}{\left({\mathrm{\mu}}_{{\mathrm{j}}_{\mathrm{s}}}\left(\mathrm{t}\right)\right)}^2-{\left(\frac{1}{\mathrm{n}}\sum_{{\mathrm{j}}_{\mathrm{s}}=1}^{{\mathrm{J}}_{\mathrm{s}}}{\mathrm{n}}_{{\mathrm{j}}_{\mathrm{s}}}{\mathrm{\mu}}_{{\mathrm{j}}_{\mathrm{s}}}\left(\mathrm{t}\right)\ \right)}^2} \end{equation*}

We formulate a mixture-based likelihood for time-varying GSDs at all significant SNPs, expressed as


(7) \begin{equation*} \mathrm{L}\left(\mathbf{g}\right)=\prod_{\mathrm{s}=1}^S\left[{\pi}_1{\mathrm{f}}_1\left({\boldsymbol{g}}_{\mathrm{s}};{\mathbf{u}}_1,\Sigma \right)+\dots +{\pi}_L{\mathrm{f}}_{\mathrm{L}}\Big({\boldsymbol{g}}_{\mathrm{s}};{\mathbf{u}}_L,\Sigma \Big)\right] \end{equation*}

The supplementary text describes specific procedures for functional clustering. We assume that QTLs from the same modules have similar biological functions. We perform gene enrichment analysis of a set of QTLs from each module using the Bulk GO Annotation Tool described in the Arabidopsis Information Resource *(*TAIR) (http://arabidopsis.org). We annotate QTLs from each module in terms of molecular function, biological process, and cellular component.

## Supplementary Material

Web_Material_uhae172
