# Supplementary material for: A nested reciprocal experimental design to map the genetic architecture of transgenerational phenotypic plasticity
Source: Hortic Res. 2024 Jun 25;11(8):uhae172. doi: 10.1093/hr/uhae172 (PMC11301319; doi:10.1093/hr/uhae172)
Supplement: Web_Material_uhae172 [file web_material_uhae172.zip › PPP_Sup(Rev).docx]

**Supplementary Text**

where $\pi_{l}$ is the proportion of mixture *l* (l = *1*, …, *L*) among *S* QTLs and $\text{f}_{\text{l}}\text{(}\boldsymbol{g}_{\text{s}};\text{u}_{l}\text{,Σ)}$ is a multivariate normal density distribution with mean vector $\text{u}_{l}$ and covariance matrix $\text{Σ}$. Because time-varying GSDs may have no explicit form, we implement Legendre orthogonal polynomials (LOP) as a smoothing function. The advantages of LOP include orthogonality, sparsity, convergence, etc. It represents a polynomial solution of the following equation:

(1)

$$\text{(1-}\text{v}^{\text{2}}\text{)(}\frac{\text{d}^{\text{2}}\text{P}_{\text{r}}\text{(v)}}{\text{d}\text{v}^{\text{2}}}\text{)-2}\text{v}\text{(}\frac{\text{d}\text{P}_{\text{r}}\text{(v)}}{\text{dv}}\text{)+}\text{r}\text{(}\text{r}\text{+l}\text{)}\text{P}_{\text{r}}\text{(}\text{v}\text{)=0}$$

The generalized solution of the LOP of order *r* can be expressed as

(2)

$$\text{P}_{\text{r}}\text{(}\text{v}\text{) =}\sum_{\text{m=0}}^{M} \text{(-1}\text{)}^{\text{m}}\frac{\text{(}\text{2r-2m}\text{)!}}{\text{2}^{\text{r}}\text{m}\text{!(}\text{r-m}\text{)!(}\text{r-2m}\text{)!}}\text{v}^{\text{r-2m}}$$

where *M* is an integer expressed as *r*/2 or (*r*-1)/2 and *v* is the independent variable, i.e., the half-marker coordinate in the curve. Let $\text{P}_{\text{l}r_{l}}\text{(}\text{t}\text{) = (}\text{P}_{\text{l}\text{0}}\text{(}\text{t}\text{),}\text{ P}_{\text{l}\text{1}}\text{(}\text{t}\text{),…,}\text{ P}_{\text{l}r_{l}}\text{(}\text{t}\text{)) }$denote the LOP of order *r_l_* on the *l*th SNP module. The mean effect of the *l*th SNP module can be expressed as $\text{P}_{\text{l}r_{l}}\text{(}\text{t}\text{)}\alpha_{l}$, where $\alpha_{l}$ is the basis value of the *l*th SNP module. In the *r*-order LOP, the order *r* directly affects the effectiveness of the data fit. The selection criterion BIC is used to determine the optimum order. Under order *r*, the BIC is calculated as follows:

(3)

$$\text{BIC }\text{= -2ln}\text{L}\text{(}\hat{\text{β}}\text{∣}\text{r}\text{)+2dimension(}\hat{\text{β}}\text{∣}\text{r}\text{) }$$

where $\text{(}\hat{\text{β}}\text{|}\text{r}\text{)}$ is the maximum likelihood estimate of the *r*-order LOP parameters and *dimension*$\text{(}\hat{\text{β}}\text{|}\text{r}\text{)}$ is the number of parameters at *r*-order. The covariance matrix of $\text{f}_{\text{l}}\text{(}\boldsymbol{g}_{\text{s}};\text{u}_{l}\text{,}\text{Σ)}$ is modeled by SAD(1).

Kim et al. (2008) implemented the EM algorithm to estimate the parameters in the mixture model . By assuming different numbers of mixtures, we estimate the MLEs of the model parameters under this assumption. The BIC criterion is used to determine an optimal number of QTL modules. After this number is known, we calculate the posterior probability of a QTL that is attributed to a given module conditional upon its GSD value. Using these posterior probabilities, we assign a QTL to its optimal module.


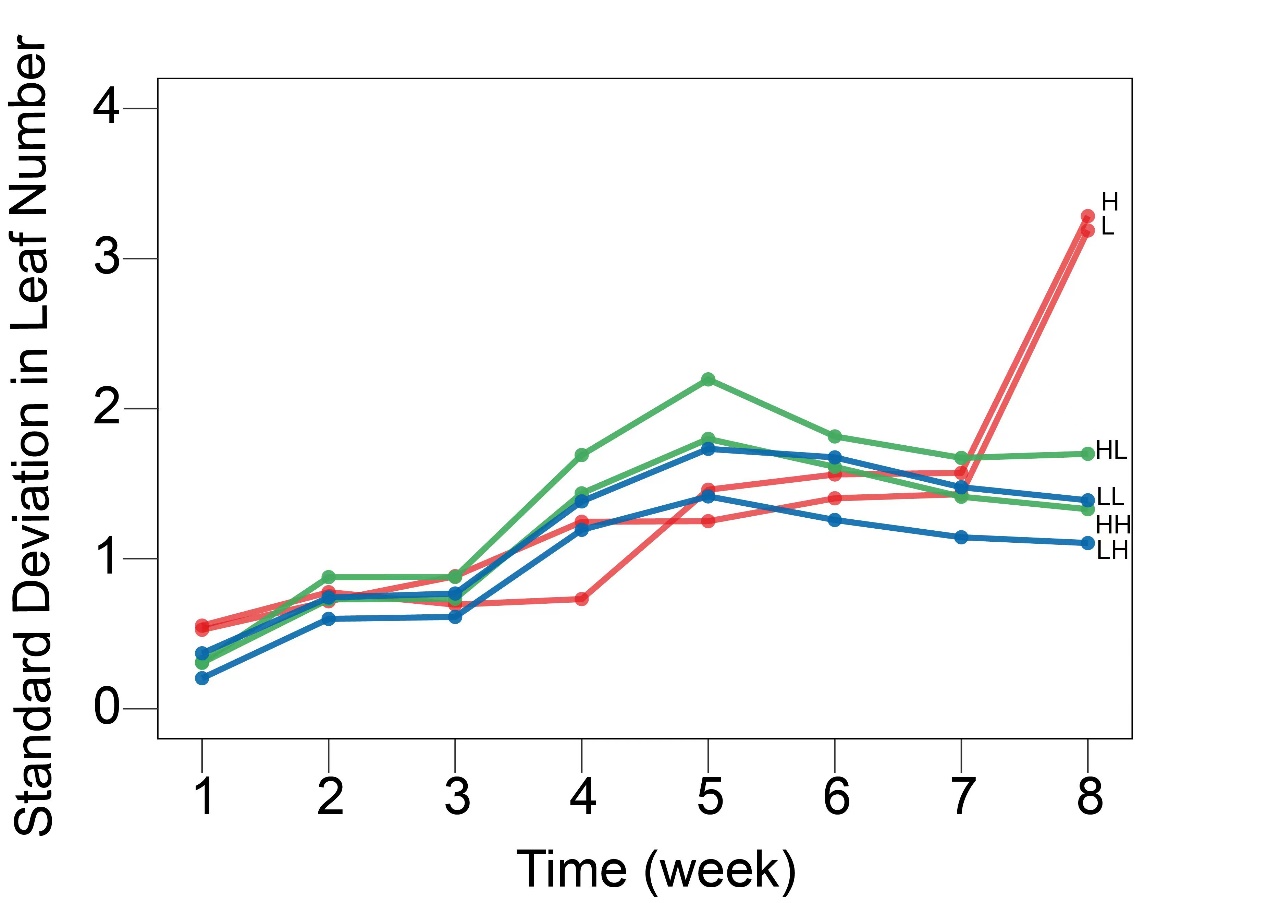


**Figure S1.** Temporal pattern of standard deviations for leaf number among RILs under six treatments.


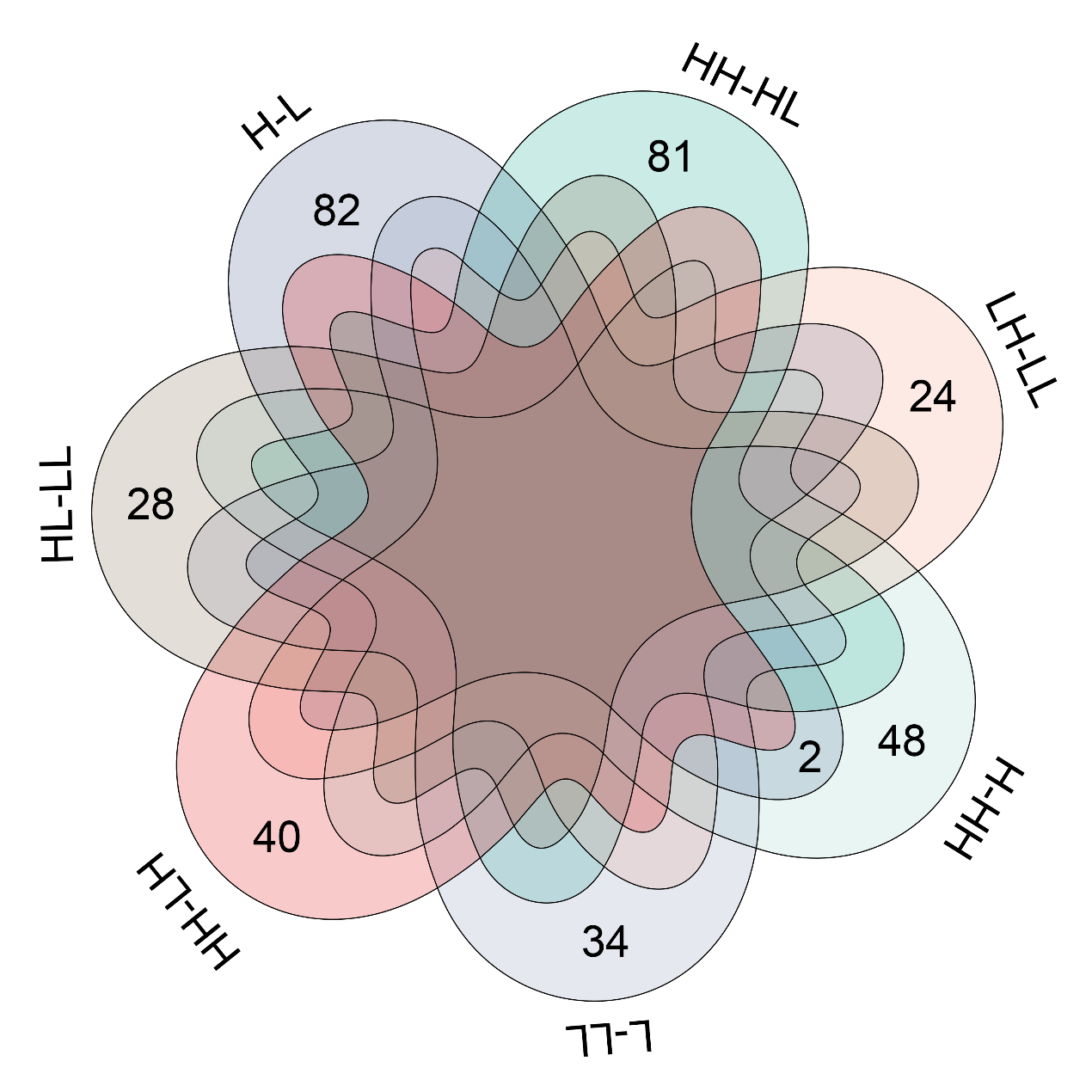


**Figure S2.** Venn diagram of the number of significant QTLs for different types of phenotypic plasticity.


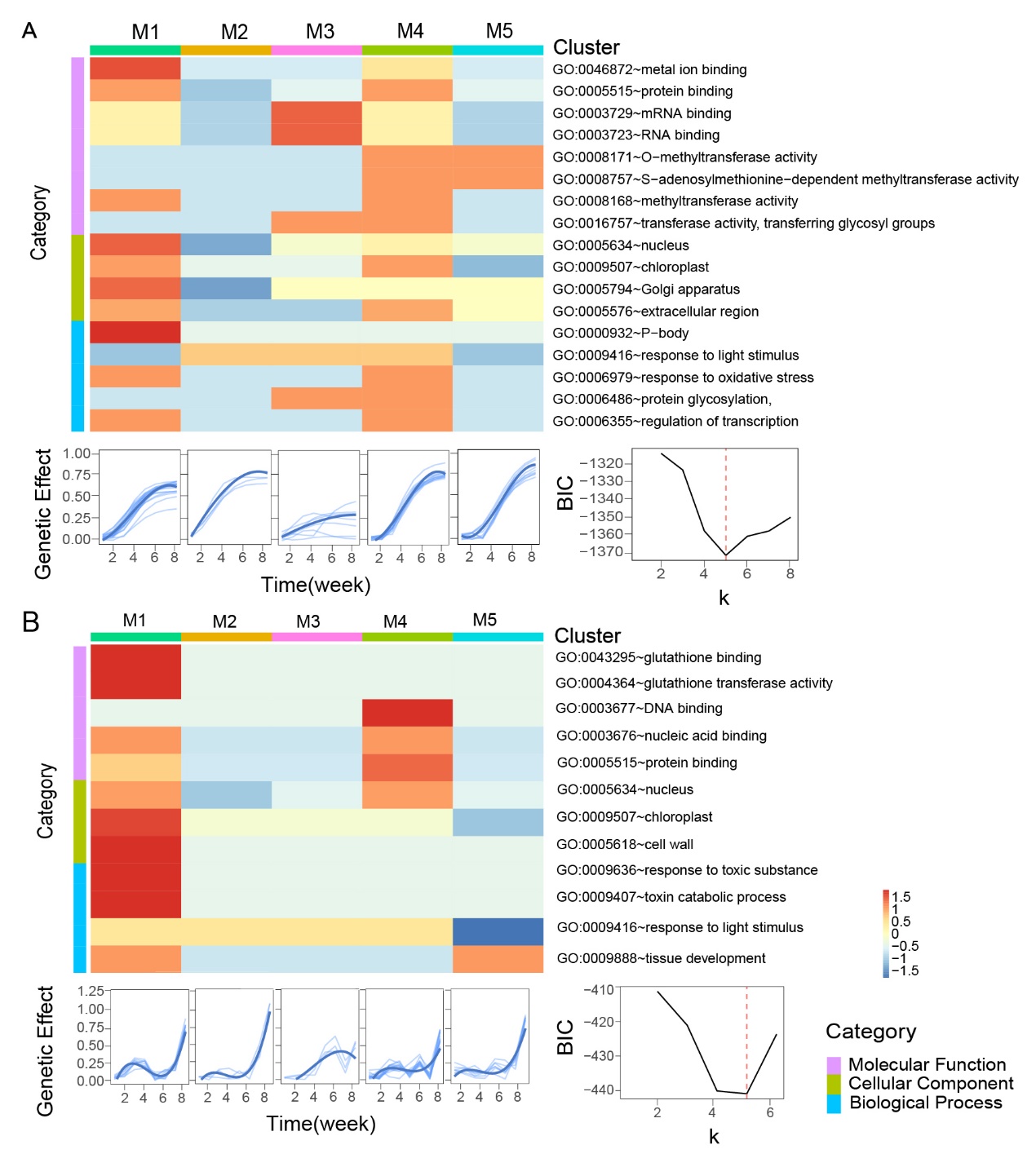


**Figure S3.** Modularity analysis of TPP QTLs. (A) Five QTL modules for TPP_H derived from the H maternal environment including the heat map of gene functions, time-varying pattern of mean gene effects for each module, and the BIC plot finding an optimal number of QTL modules. (B) Five QTL modules for WPP_L derived from the L maternal environment, including the heat map of gene functions, time-varying pattern of mean gene effects for each module, and the BIC plot finding an optimal number of QTL modules.


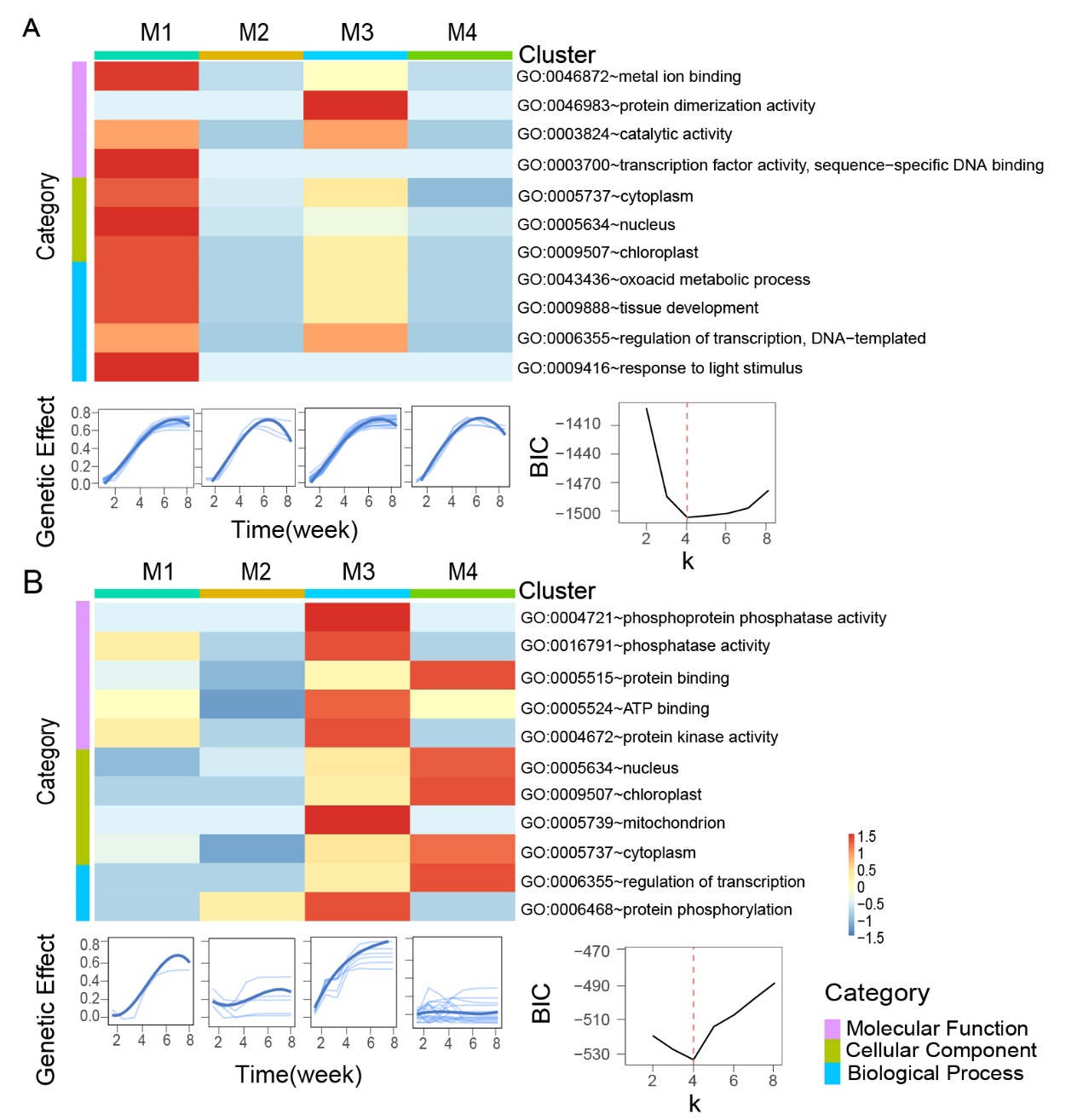


**Figure S4.** Modularity analysis of MPP QTLs. (A) Four QTL modules for MPP_H expressed in the H offspring environment including the heat map of gene functions, time-varying pattern of mean gene effects for each module, and the BIC plot finding an optimal number of QTL modules. (B) Four QTL modules for MPP_L expressed in the L offspring environment including the heat map of gene functions, time-varying pattern of mean gene effects for each module, and the BIC plot finding an optimal number of QTL module.
